# Redesigning Pharmacy to Improve Public Health Outcomes: Expanding Retail Spaces for Digital Therapeutics to Replace Consumer Products That Increase Mortality and Morbidity Risks

**DOI:** 10.3390/pharmacy12040107

**Published:** 2024-07-12

**Authors:** Grzegorz Bulaj, Melissa Coleman, Blake Johansen, Sarah Kraft, Wayne Lam, Katie Phillips, Aarushi Rohaj

**Affiliations:** 1 Department of Medicinal Chemistry, College of Pharmacy, University of Utah, Salt Lake City, UT 84112, USA; 2 College of Pharmacy, University of Utah, Salt Lake City, UT 84112, USA; 3 Independent Researcher, Salt Lake City, UT 84112, USA; 4 The Spencer Fox Eccles School of Medicine, University of Utah, Salt Lake City, UT 84112, USA

**Keywords:** pharmacy care, drug store, soda, liquor, liability, negligence, mHealth, smartphone app, therapeutic video games, digital interventions, patient empowerment

## Abstract

United States healthcare outcomes, including avoidable mortality rates, are among the worst of high-income countries despite the highest healthcare spending per capita. While community pharmacies contribute to chronic disease management and preventive medicine, they also offer consumer products that increase mortality risks and the prevalence of cardiovascular diseases, diabetes, cancer, and depression. To resolve these contradictions, our perspective article describes opportunities for major pharmacy chains (e.g., CVS Pharmacy and Walgreens) to introduce digital health aisles dedicated to prescription and over-the-counter digital therapeutics (DTx), together with mobile apps and wearables that support disease self-management, wellness, and well-being. We provide an evidence-based rationale for digital health aisles to replace spaces devoted to sugar-sweetened beverages and other unhealthy commodities (alcohol, tobacco) that may increase risks for premature death. We discuss how digital health aisles can serve as marketing and patient education resources, informing customers about commercially available DTx and other technologies that support healthy lifestyles. Since pharmacy practice requires symbiotic balancing between profit margins and patient-centered, value-based care, replacing health-harming products with health-promoting technologies could positively impact prevention of chronic diseases, as well as the physical and mental health of patients and caregivers who visit neighborhood pharmacies in order to pick up medicines.

## 1. Introduction

**Figure 1:**
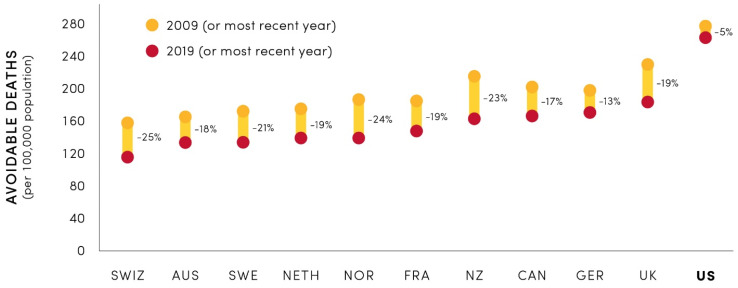
Despite the highest healthcare spending per capita, the number of avoidable deaths is higher in the US (marked in bold font for emphasis) as compared to other high-income countries, while 10-year reduction in avoidable mortality in the US is inferior as compared to other high-income countries. SWIZ, Switzerland; AUS, Australia, SWE, Sweden; NETH, the Netherlands; NOR, Norway; FRA, France; NZ, New Zealand; CAN, Canada; GER, Germany; UK, the United Kingdom; US, the United States. This figure was originally shown as Exhibit 8 in the Commonwealth Fund report [1], and was reproduced with permission from the publisher.

Despite the highest healthcare spending per capita, the United States (US) healthcare systems underperform compared to other high-income countries [1]. One of several performance and outcome indicators that need improvement in the US is the mortality rate [1]. As illustrated in Figure 1, the US has had a remarkably small improvement in avoidable mortality compared to 10 other high-income countries. These findings coincide with deteriorating life expectancy in the US [2,3]. According to the Centers for Disease Control and Prevention (CDC), 60% of adult Americans have at least one chronic disease [4]. In addition to the high economic burden of chronic diseases, they also are the leading causes of death, disability, and poor health in the US [4,5]. Some challenges associated with reducing chronic disease prevalence and burden are healthcare affordability, accessibility, and inequality [6,7]. 

To reduce preventable causes of premature death in the US, the CDC recommends several strategies including “environmental approaches that promote health and support healthy behaviors” [5]. As shown in Figure 1, poor outcomes of reducing preventable mortality in the US require more alignment of multiple stakeholders to promote health by creating indoor and outdoor environments that foster healthy lifestyles. With respect to pharmacy care, some examples of misalignment include the selling and price-based promotion of liquor in pharmacy stores (Figure S1) [8,9,10], while the CDC reported that “Excessive alcohol use was responsible for about 178,000 deaths in the United States each year during 2020–2021” [11]. 

Pharmacies selling health-harming food, beverages, and/or tobacco products can create ethical and professional dilemmas among pharmacists [12,13], as can be seen through several legislative efforts to ban these sales in pharmacies [14]. Even though (1) tobacco and alcohol consumption are the leading causes of preventable death in the US, and (2) the American Pharmacists Association has a policy opposing sales of alcohol and tobacco in pharmacies, many retail pharmacies continue to offer these products to their consumers [9]. 

Pharmacy practice has expanded from counting and dispensing in retail settings to working in diverse clinical and healthcare settings, including chronic disease management [15], psychosocial support [16], point-of-care testing [17], clinical diagnostics [18], awareness campaigns [19], and public health interventions [20,21]. Pharmacists have a special relationship with their patients, allowing them to better counsel and educate them not only on medicines, but also on disease prevention [20,22,23]. Driven by the emergence and clinical validation of digital health technologies, this article connects two aspects of how pharmacists in the US might improve avoidable mortality rates by (1) expanding access to digital health technologies that support chronic disease management and prevention, and (2) eliminating sales of consumer products that increase mortality risks. 

## 2. Digital Health Technologies for Improving Healthcare Outcomes

Digital health is a broad category of technologies that include digital therapeutics (DTx, mobile medical apps), mobile health (mHealth) apps, wearable devices, health IT, telehealth, and telemedicine. Digital health technologies are intended to improve healthcare outcomes through better diagnosis, management, and prevention of medical conditions. In order to advance the development and implementation of these technologies into healthcare, the US Food and Drug Administration (FDA) has established the Digital Health Center of Excellence [24]. Additional resources that promote digital health technologies include The Digital Medicine Society, The Digital Therapeutics Alliance, and the Digital.Health online platform.

DTx are mobile medical apps that received regulatory clearance as “Software as a Medical Device” (SaMD) products for the treatment, diagnosis, or prevention of medical conditions [25,26,27,28,29,30]. SaMD is defined as “software intended to be used for one or more medical purposes that perform these purposes without being part of a hardware medical device” [24]. DTx obtain the FDA’s authorization as class I or II medical devices using 510(k) clearance, exempt, or de novo regulatory pathways and can be classified into two main categories: (1) prescription digital therapeutics (PDTs) that require a prescription from a healthcare provider, and (2) over-the-counter (OTC) digital therapeutics that can be purchased online without a prescription. DTx products that pose a low risk for patients can also be marketed under the FDA enforcement discretion policy. DTx include such technologies as web-based, mobile, or virtual reality (VR) apps and video games. 

Digital health technologies, including DTx, provide clinically meaningful benefits for diverse chronic medical conditions, such as low back pain, attention deficit hyperactivity disorder (ADHD), post-traumatic stress disorder (PTSD), diabetes, insomnia, migraine, substance use disorder, heart failure, depression, anxiety, rheumatoid arthritis, cancer, chronic obstructive pulmonary disease (COPD), irritable bowel syndrome (IBS), neurodegenerative and cardiovascular diseases (CVDs), etc. [27,31,32,33,34,35,36]. As illustrated in Table 1, RelieVRx is an example of an FDA-authorized PDT that uses a VR system to reduce the severity of chronic low back pain (CLBP) [37,38,39]. Examples of OTC DTx and PDT are EndeavorOTC and EndeavorRx, video game apps that can improve focus and cognitive functions in adults and pediatric patients with ADHD, respectively [40,41,42]. According to marketing from Akili Interactive, playing these therapeutic video games in two “six-week doses” separated by a one-week interval can lead to the best outcomes [42]. The WellDoc digital health platform supports self-management of cardiometabolic diseases, including prescription and OTC DTx for diabetes types 1 and 2. 

Other categories of digital health technologies are wearables, neurotech, and wellness apps, intended to promote healthy lifestyles and improve health outcomes [43,44,45]. For example, physical activity trackers such as Apple Watch, Fitbit, Garmin, and Galaxy Watch can improve physical activity, mental health, and other health outcomes [46,47,48,49]. Incorporating Fitbit into daily lifestyle interventions significantly lowered the risks associated with sleep apnea, obesity, depression, diabetes, and hypertension [50]. Some of these technologies are at the nexus of medical devices and consumer health products. For example, Samsung Electronics received FDA authorization for the Samsung Health Monitor app to detect signs of obstructive sleep apnea via the Galaxy Watch [51]. Apple Watch combined with the StrivePD app received FDA clearance for monitoring symptoms of Parkinson’s disease [52]. The Oura ring is a consumer product worn on the finger intended to work with an app to monitor heart rate, body temperature, blood oxygen, sleep and physical activity patterns, and women’s health. The Oura ring can accurately measure sleep time, sleep onset latency, time spent in deep and light sleep, and wake after sleep onset [53]. Digital health products also include non-invasive vagal nerve stimulators, such as Nurosym, or Hinge Health Enso, a wearable that delivers high-frequency impulse therapy for pain [54]. Table 1 provides examples of DTx and other digital health and wellness technologies that can improve health outcomes.

**Table 1:** Examples of digital health technologies intended to improve health outcomes. PDT, prescription digital therapeutic; OTC, over-the-counter; AI, artificial intelligence.

Products	Type of Product	Healthcare Outcomes	References
RelieVRx	FDA-cleared virtual reality system as PDT intended for treatment of chronic low back pain	Long-term, non-opioid pain reduction in adults with moderate to severe chronic low back pain	[39,55,56,57]
Welldoc App, BlueStar	FDA-cleared PDT and OTC DTx mobile apps that provide support and digital coaching for patients with diabetes, prediabetes, heart failure, and hypertension	Diabetic patients experienced a statistically significant improvement in hemoglobin A1c levels	[58,59]
EndeavorOTC, EndeavorRx	OTC DTx and PDT mobile game designed to treat and improve attention deficit hyperactivity disorder (ADHD) symptoms in children and adults using a series of video games	Improvements in attention, multi-tasking, and cognitive function for adults with ADHD. PDT version was also effective for pediatric ADHD	[41,60,61]
Daylight by Big Health	CBT-based mobile app marketed for generalized anxiety disorder under the FDA enforcement discretion	Long-lasting improvement of generalized anxiety	[62,63]
Sleepio	Digital CBTi intervention for insomnia marketed under FDA enforcement discretion	Improves irregular sleeping patterns. Has shown effectiveness in improving sleep quality in working adults and adolescents	[64,65,66,67]
Rejoyn	FDA-cleared adjunct PDT for adults with major depressive disorder (MDD)	Improvement of depressive symptoms for MDD patients who take antidepressants	[68]
Samsung Galaxy Watch	FDA-cleared wearable for sleep tracking	Supports management and monitoring of sleep, sleep score, and time asleep	[69]
Oura ring	Consumer wearable for monitoring sleep and general health	Supports healthy lifestyle by monitoring sleep, physical activity, heart rate, and overnight blood oxygen level	[53,70]
Fitbit	Consumer activity tracker includes an FDA-cleared detection of irregular heart rhythms as signs of atrial fibrillation	Monitoring and promoting physical activity, heart health, and sleep to support healthy lifestyles	[50,71,72,73,74]
Muse EEG headband	Consumer wearable, EEG-based biofeedback headband. Marketed as “at-home biofeedback training” device	Monitoring and improving mental well-being, obsessive–compulsive disorder (OCD) symptoms, and cognitive functions	[75,76,77,78,79,80]
Nurosym	Non-invasive vagal neurostimulation device, certified medical device in Europe	Improved heart rate variability, reduction of postural orthostatic tachycardia syndrome (POTS) symptoms	[81,82]
Nerivio	FDA-cleared, non-invasive neurostimulation wearable targeting inhibitory pain pathways	Provides pain relief, migraine treatment and prevention	[83,84,85,86,87]

Digital health technologies are being recognized for enhancing pharmacy care [88,89,90,91,92,93,94]. There are apparent benefits of integrating DTx with drug-based therapies, such as better therapy adherence and outcomes [31,95,96,97,98,99]. For example, adjunct DTx reSET-O improves cost-effectiveness and treatment outcomes of buprenorphine for opioid use disorder [98,99,100,101]. Connecting insulin-delivery devices with continuous glucose monitoring systems offers better diabetes management [102,103,104]. Mobile apps can also improve medication adherence in people with diabetes [105,106]. The FDA draft guidelines on “prescription drug use-related software” support the development of drug+digital combination therapies [31,107]. We recently reviewed how digital therapeutics can improve the effectiveness of pharmaceutical drugs and biologics using the drug–device combination product approach [31]. Rapid developments in applications of artificial intelligence (AI) to precision medicine support broader adoption of digital technologies for better healthcare outcomes [108]. 

## 3. Redesigning Retail Pharmacy Spaces for Digital Health Technologies

The physical environment and professional image of community pharmacy spaces impact both visiting customers and working pharmacy staff [109]. Many community pharmacies share a similar structure: a pharmacy counter with a few windows open for patients to drop off and pick up their prescriptions and a store section containing aisles of OTC medicines, dietary supplements, consumer health products, and various snacks and beverages. The general layout of most pharmacies funnels patients and other customers through the aisles while guiding them to the counter to pick up medicines. If their prescription medicines are not ready, some patients wander the pharmacy, browsing the available products. Patients waiting in line or in the waiting area for their prescriptions might occupy their time by looking at OTC medicines and all other products that are displayed in adjacent aisles. 

**Figure 2:**
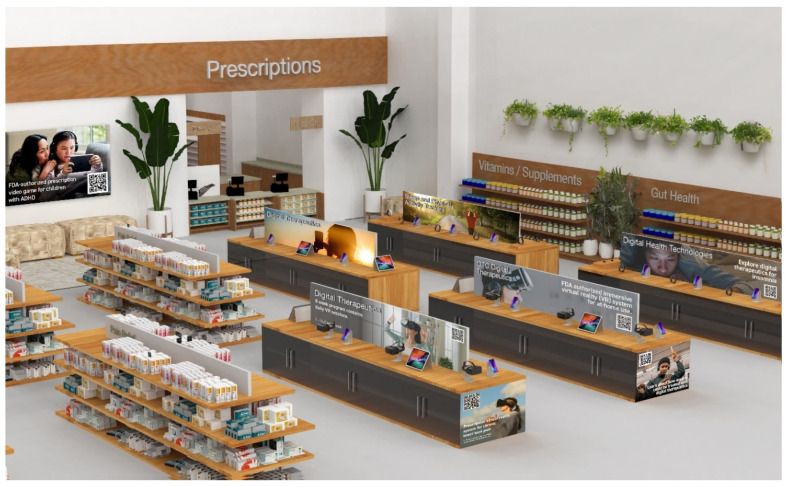
Rendering of a retail pharmacy space with aisles dedicated to selling and marketing diverse digital health technologies ranging from OTC and prescription digital therapeutics to health-promoting consumer wearables and wellness mobile apps.

Herein, we describe opportunities to redesign aisles to offer digital health technologies to those who visit community pharmacies. As illustrated in Figure 2, such modifications to a retail space would require changing the standard layout of the pharmacy space. Digital health aisles would ideally be situated close to the pharmacy counter. The aisles containing the digital therapeutic products may be organized by disease state, similar to over-the-counter medicines. For example, a digital health aisle for diabetes would showcase products that can improve glycemic control and diabetes management, including glucose monitoring apps, insulin titration and delivery apps, nutrition and physical activity apps [110,111,112], and even therapeutic video games [113]. To optimize the consumer experience and education about these new technologies, interior design and displays can be similar to those used in consumer electronics retail shops (e.g., Best Buy and Apple Stores). Pharmacy customers could access demos and purchase consumer-grade and OTC digital health apps by scanning associated QR codes that direct them to specific websites (analogous to the affiliate marketing mechanism). Some aisles may have digital display screens or VR headsets for customers to use. Here, customers can use the headsets to experience some digital therapeutics, such as RelieVRx, and test whether or not the product is right for them. Mobile applications such as EndeavorOTC could be advertised by allowing customers to try out select games on the display screen. These interactive and entertaining displays would serve as a way to engage customers with a product they might have otherwise ignored [114].

Other aisles could be dedicated to the prevention and treatment of diverse mental health conditions, chronic pain, arthritis, and other chronic diseases. In addition to diverse DTx products, pharmacies can offer digital health and consumer products that support disease self-management and healthy lifestyles. Fitness apps and activity trackers can serve as patient empowerment tools for people living with chronic conditions to exercise regularly and improve sleep hygiene [44,115,116]. Consumer-grade wearables, such as the Muse EEG headband, offer a means for biofeedback and mindfulness-based interventions to improve mental health [79,117,118,119]. Digital health aisles can not only serve as a method of promoting and selling DTx, but also provide patients with an opportunity to learn about diverse non-pharmacological approaches to disease management and prevention. To offer digital health products that are of the highest quality with respect to clinical validation, effectiveness, functionality, user experience, cybersecurity and privacy protection, retention, and other important features, pharmacies may collaborate with appropriate professional associations such as the Digital Therapeutics Alliance, the Digital Medicine Society, and the Digital.Health platform. 

## 4. Digital Health Aisles Support Patient Education and Marketing

Since community pharmacies are uniquely positioned to promote healthy lifestyles [120], introducing digital health aisles into the pharmacy space would facilitate educating patients and all visiting customers on available digital health technologies. Pharmacy lines are known to be quite long (for example during seasonal flu seasons), and posters can offer patients information to interact with to occupy their time. Health education and promotion posters and boards have been effectively used in the waiting rooms of medical offices in an attempt to educate patients while they wait [121,122]. Posters are also used in community pharmacies to promote vaccinations [123]. Studies show that posters, displays, and leaflets positively affect health education, provided they are in a location where the patient will have an extended amount of time to look at them and visit regularly [124,125]. As illustrated in Figure 2, posters and displays across the digital health aisles can provide a catching visual aid that will deliver patient education and marketing of digital health technologies. Allowing the patient to see the poster and view it for an extended period every time they come into the pharmacy will increase the number of times they think about the DTx and will prompt the patient to ask more about it [124]. 

**Figure 3:**
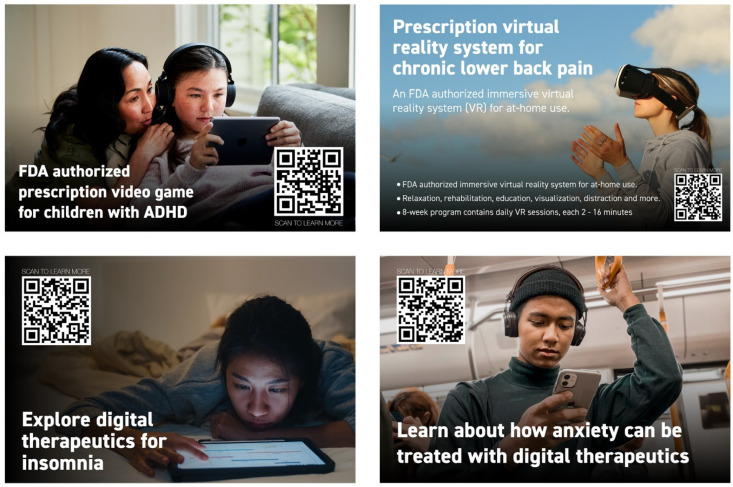
Examples of posters dedicated to patient education and marketing of digital health technologies in retail pharmacies. Such posters can be located as wall posters or display boards across the pharmacy space.

Wall posters, electronic and printed displays, and leaflets are a great way to impact consumer buying behaviors [126], as well as to educate them about the diversity and benefits of DTx and other digital health technologies. This “marketing as education” approach in retail pharmacies would likely accelerate dissemination of the related knowledge among health professionals [127,128,129]. Examples of such posters and displays are shown in Figure 3. These posters provide brief information about DTx such as EndeavorOTC for adult ADHD, RelieVRx for chronic low back pain, and Big Health apps for insomnia and anxiety.

Placing digital health aisles close to the pharmacy service counter will encourage patient interaction with DTx products and allow pharmacists to engage with patients showing interest in specific displays. This would allow pharmacists to provide recommendations for (1) patients who may be taking multiple medicines for one disease state and would benefit from using a DTx to help manage their condition, (2) elderly patients who look for digital tools to improve their cognitive functions and physical health, (3) caregivers of children living with chronic conditions, and (4) caregivers of people living with cognitive disabilities. Providing opportunities for a pharmacist to educate often skeptical patients and recommend digital interventions will increase awareness and use of DTx. As elaborated later, community pharmacies that disseminate evidence-based information about digital health to their consumers could become “digital health education hubs”. From the public health perspective, pharmacies may prioritize patient education and marketing of digital health technologies that support cardiovascular and metabolic health, chronic pain management, smoking cessation, substance use disorder management, and management of other mental health conditions. 

Pharmacists’ expertise in counseling patients on their medicines and monitoring therapy presents a timely opportunity to expand the digital health skills of pharmacy staff [130,131,132,133]. Since telehealth and digital health products such as continuous glucose monitoring devices already exist, pharmacists can deepen their abilities to interpret and apply their knowledge to help patients with digital interventions [88]. Therefore, educating healthcare professionals, such as pharmacists, is an important part of implementing DTx into healthcare [130]. Providers need to be knowledgeable about DTx and other digital health technologies so that they can effectively explain their benefits, how to use them, and how they work. Ultimately, educating healthcare providers on DTx means they will be well equipped and qualified to educate patients that will benefit the most from using DTx and increase positive outcomes in patients’ lives [134]. 

## 5. Pharmacies Selling Consumer Products That Increase Mortality Risks

Despite challenges for the US healthcare systems to reduce chronic diseases, some retail pharmacies sell consumer products that increase risks for chronic diseases and premature death. In doing so, these pharmacies enable health-harming industries to target Americans who visit them [135,136,137]. For example, retail pharmacies sell alcohol and tobacco products that are recognized as the leading cause of preventable death in the US [9]. Given that over 480,000 people in the US die from cigarette smoking each year [138], offering tobacco products in pharmacies is a stark contradiction to the efforts of healthcare professionals who work towards saving lives [139,140]. While CVS Health discontinued selling tobacco products in 2014 [141], other retail pharmacies, e.g., Walgreens, continue to offer tobacco products. It is noteworthy that pharmacies in the US are engaged in cigarette promotions including discount pricing, multi-pack, and cross-product promotions [142]. This study also found that “Cigarette promotions were observed in 94.0% of pharmacies, more than any other retailer type (e.g., convenience stores: 82.0%, tobacco stores: 77.0%)” [142], while other studies show positive effects of pharmacist-delivered smoking cessation interventions [143,144,145,146].

Similarly, despite the CDC reporting that about 178,000 Americans died in a year (2020–2021 data) from alcohol-related causes [11], many retail pharmacies sell alcoholic beverages including spirits, wine, and beer [8,10] (see also Figure S1 in the Supplementary Materials). According to the National Institute of Health, over 10% of people age 12 and older in the US have alcohol use disorder (AUD) [147]. While some retail pharmacies sell alcoholic beverages, clinical pharmacists are involved in screening patients for alcohol use disorder and facilitating access to AUD treatments [18,148]. Alcohol is classified as a dependence-producing substance and Group 1 carcinogen, and is recognized for causing several types of cancer and its contribution to cancer-related preventable deaths in the US [149,150]. Alcohol-related deaths significantly contribute to total deaths in the US [151].

In addition to selling alcohol and tobacco, some US pharmacies also sell and endorse (through promotional pricing) sugar-sweetened beverages (SSBs) [13,152,153,154], creating a potential negligence to protect their customers, given a growing body of evidence that SSB consumption can increase both mortality and morbidity risks [155,156,157,158,159,160,161,162,163]. National pharmacy chains can reach SSB sales of estimated 100 million transactions per year (Figure S2, Supplementary Materials). Several longitudinal studies show that higher consumption of SSBs is associated with higher risks for premature death due to cardiovascular diseases and cancer, as summarized in Table 2. Malik and colleagues found that consuming two or more SSBs per day increased the risk of death from cardiovascular disease (CVD) by 31% [156]. Higher SSB consumption is positively associated with higher risks of developing cancer and cancer-related mortality [164]. Of equal importance to public health are correlations between SSB intake and obesity [165], diabetes [166,167,168], and mental health [169,170,171,172]. Alcaraz and colleagues evaluated 40 different articles and 18 different models suggesting that policy intervention is crucial to decrease the 184,000 deaths worldwide annually attributed to consumption of SSBs [158]. 

**Figure 4:**
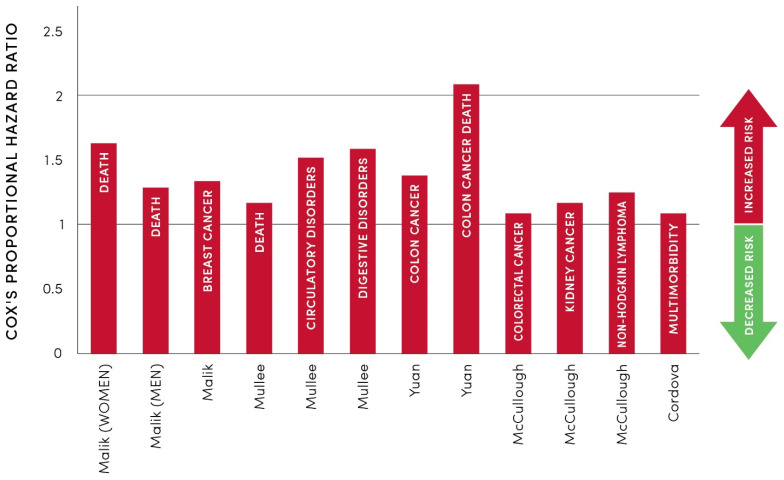
Health-harming properties of sugar-sweetened beverages expressed as Cox’s hazard ratios (CHRs). CHR values above 1 indicate increased risks for premature death or morbidities when drinking more than two SSBs daily when compared to the control groups. Detailed data used for this graph are shown in Table S1 in the Supplemental Materials.

To further spotlight the relationships between the consumption of SSBs and their health-harming effects, Figure 4 illustrates data from five different studies that used Cox’s proportional hazard ratio (CHR) to investigate the impacts of drinking more than two SSBs per day when compared to the control group of drinking one SSB or less per month (see also Table S1 in the Supplementary Materials). It is apparent that higher SSB consumption increases the risks of premature death, digestive and circulatory disorders, and various cancers. This is of particular importance to the 96 million Americans who are prediabetic, 38 million Americans who live with diabetes [178], and 127 million Americans who live with CVDs [179]. 

While negatively impacting mortality, addiction, and the obesity epidemic, distributing SSBs, alcohol, and tobacco products in community pharmacies (1) defies the commitment to improving healthcare outcomes; (2) suggests a failure to be more proactive in protecting vulnerable individuals from preventable deaths, as reminded by the national opioid settlement agreement signed by three major chain pharmacies, CVS, Walgreens, and Walmart, in 2022 [180,181,182]; (3) contributes to the negative environmental impact of healthcare and pharmaceutical industries [183,184,185], and (4) may create a mistrust between publicly traded chain pharmacies, shareholders, and the public with respect to ESG (Environmental, Social, and Governance) reporting on making people and communities healthier. From the “good faith” perspective, these aforementioned aspects are even more applicable to pharmacies owned by healthcare companies (e.g., CVS Health Corporation, Woonsocket, Rhode Island) that also offer healt h insurance through managed healthcare companies (e.g., Aetna, Hartford, Connecticut), while owning pharmacy benefit management companies (e.g., CVS Caremark, Woonsocket, Rhode Island) that profit from selling prescription drugs. 

In May 2024, CVS Pharmacy launched “the WellMarket™” portfolio of consumer health-focused beverages and food, resonating with opportunities for voluntary changes in chain pharmacy stores to improve the food environment [13]. In June 2024, the Milken Institute published the report “Catalyzing Action for Pharmacist-Provided Food Is Medicine Care” describing a rationale and actionable recommendations to implement the Food Is Medicine program delivered through local pharmacies [186]. The National Association of Chain Drug Stores also promotes health and wellness innovation in pharmacies [187]. Involvement of patient advocacy groups such as the American Heart Association and the American Diabetes Association, as well as professional groups such as the American Pharmacists Association and the American Public Health Association, can further accelerate the transformation of pharmacies towards replacing health-harming beverages and ultraprocessed food with health-promoting technologies. 

## 6. Transforming Pharmacies to Improve Healthcare and Public Health Outcomes

The commercial determinants of health require pharmacies to balance profitability, social responsibility, and value-based care [188,189,190]. An example of profit-driven practices is the contribution of pharmacy benefit managers to the high prices of prescription drugs [191,192]. Rising healthcare costs and the economic burden of chronic diseases prompted some innovative pharmacy initiatives, e.g., “Flip the Pharmacy” practice transformation [193,194,195], embracing digital health [196,197], and a pharmacist-provided “Food Is Medicine” care program [186]. To further address the contributions of pharmacists to improve health outcomes, herein we reason that (1) selling and marketing digital health technologies in retail pharmacies may improve both therapies and prevention of chronic diseases, and (2) eliminating sales of sugar-sweetened beverages may improve prevention of chronic diseases and mortality. 

Eliminating sales of SSBs and other health-harmful products in profit-driven pharmacies is a challenge that would require the same corporate social responsibility motives that led to the elimination of tobacco products from CVS Pharmacy chains in 2014 [190]. Point-of-sale warnings about the harmful effects of beverages are unlikely to change consumers’ awareness, as shown by alcohol–cancer association labels via Proposition 65 policy in California [198]. Scaling up and expanding the scope of tobacco-free pharmacy law would require coordinated efforts of all stakeholders [14,199,200]. Strict liability and negligence cases against pharmacies selling unsafe food that can cause illness and premature death would require multiple plaintiffs to prove direct causation or show that SSBs are “unreasonably dangerous”. Promoting health-harming beverages in pharmacies can be interpreted as deceptive practices targeting vulnerable customers who trust pharmacists as healthcare professionals helping them to improve their health. Therefore, to protect consumers, the Federal Trade Commission may foster self-regulation of pharmacies through advisory opinions suggesting ceasing promotional pricing of SSBs and alcohol. SSB taxes intended to improve public health by reducing SSB consumption can lead to higher prices and lower sales of these beverages [201,202,203]. However, these price-based interventions are implemented only in several cities across the US. It is important to emphasize that both the tobacco-free pharmacy law and the discontinuation of tobacco sales by CVS Pharmacy resulted in positive public health outcomes [141,204,205,206]. 

**Figure 5:**
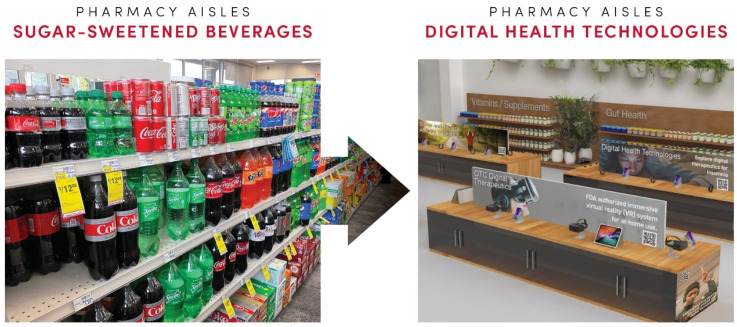
Transforming retail pharmacy environment by replacing sales and marketing of health-harming products (sugar-sweetened beverages, alcohol, and tobacco) with health-promoting digital technologies.

As illustrated in Figure 5, we propose transforming retail pharmacy practice by trading health-harming products with health-promoting digital technologies. While redesigning pharmacy spaces applies to retail pharmacies, offering digital health technologies in online pharmacies is also viable. These perspectives are already reflected in such developments as Walgreens partnering with digital health companies, e.g., Hinge Health, a digital care company for musculoskeletal conditions, Propeller Health, a precision digital care platform, and Sanvello, a mental health app marketed for stress, anxiety, and depression [207]. Benefits for digital health companies to distribute their products through pharmacies are (1) endorsement of their technologies being sold through healthcare professionals, (2) increased brand awareness and marketing through over 30,000 chain pharmacy stores (and more through independent community pharmacies), and (3) increased public awareness facilitating broader adoption of their technologies in healthcare and the general public. To increase sales, pharmacy chains can develop consumer-centered omnichannel marketing and distribution for digital health technologies that are integrated with the use of prescription and OTC medicines. Opportunities to embrace digital health in pharmacies can also be complemented by scaling up telepharmacy services and point-of-care testing [196,208,209,210,211,212,213]. These “new” products and services could mitigate revenue and profit margin changes resulting from discontinuing sales of SSBs, alcohol, and tobacco products. 

It was estimated that CVS Health lost approx. USD 2B in yearly revenues by discontinuing tobacco sales [190,214]. While revenue losses from discontinuing sales of SSBs, alcohol, and tobacco in pharmacies are hard to evaluate due to the lack of sales data, trading health-harming commodities with health-promoting digital health technologies would lessen the financial impact of such corporate decisions. It is noteworthy that the US market size for digital health varies in estimates from USD 89B to USD 133B (2022 data) and is projected to grow substantially [215,216]. On the other hand, sales of SSBs have been steadily declining [217]. 

The value proposition for replacing health-harming products with digital health technologies can be driven by joint venture partnerships between pharmacies and accountable care organizations. For example, drug-based managements of cardiovascular conditions and diabetes can be improved with less consumption of sugar-sweetened beverages, while Ozempic, naltrexon, and antidepressants work better when patients are empowered to increase physical activity, rather than when being exposed to counterproductive cues such as ultraprocessed foods and alcohol products offered in pharmacies. Since the FDA has enabled development of “prescription drug use-related software” products, pharmacies that quickly evolve to distribute digital health technologies may gain competitive advantage over retail pharmacies that retain the current portfolio of products and services. 

In addition, pharmacies may boost their sales and revenues through cross-promotions of digital health technologies together with OTC medicines (e.g., low-dose aspirin for the primary prevention of heart attack and stroke, or NSAID analgesics for chronic pain), vaccinations (e.g., the shingles vaccine), dietary supplements (e.g., multivitamin–mineral supplements and vitamin D), and in vitro diagnostic products (e.g., at-home testing kit Lucira^®^ for the simultaneous detection of COVID-19 and flu A/B viruses). Offering fitness trackers, sleep monitoring wearables, and wellness apps in pharmacies may increase their revenues from the global wellness market estimated USD 1.5 trillion [218]. 

To further increase customer engagement, satisfaction, and retention, community pharmacies may become known as “digital health education hubs”, where clients obtain evidence-based knowledge and the latest updates about the benefits of health-promoting digital technologies. Such digital health education centers can be created in collaboration with the Digital Therapeutics Alliance, the Digital Medicine Society, and the Digital.Health platform, while being sponsored by integrated health systems, such as Intermountain Health, Kaiser Permanente, Trinity Health, the Department of Veterans Affairs, Mayo Clinic, Cleveland Clinic, HCA Healthcare, and Universal Health Services, as well as by biotech, pharmaceutical, and pharmacy benefit management companies through their Environmental, Social, and Governance (ESG) investment arms. 

Smaller community pharmacies that do not belong to major chains could benefit from lawmakers increasing the budget for the CDC’s chronic disease prevention and health promotion programs that would financially incentivize transformation of their retail spaces towards digital health technologies, while eliminating sales of health-harming products. There is also a potential role for philanthropic organizations that support health equity, public health, and biomedical research, such as the Robert Wood Johnson Foundation, Howard Hughes Medical Institute, Bill and Melinda Gates Foundation, and others, to expand their programs towards digital health promotion through grants awarded to local pharmacies. 

## 7. Limitations and Challenges

While this perspective article describes opportunities and benefits of selling digital health technologies in retail pharmacies instead of health-harming products such as SSBs, alcohol, and tobacco, we acknowledge the real-world limitations of this work. For example, digital health technologies are rapidly evolving, presenting retailers with the challenges of selling products that may be discontinued or require frequent software updates to optimize their effectiveness. Since distribution channels and reimbursement processes for DTx have been evolving [219], payment strategies and affiliate marketing require complex financial arrangements between digital health companies and wholesale/retail sellers. 

DTx and other digital health technologies are relatively new, creating a skepticism among healthcare professionals about the level of clinical validation and effectiveness as compared to other therapeutic modalities. Barriers to adoption of digital health technologies also include digital health literacy, health literacy, and the socioeconomic status of consumers [220,221,222], thus potentially discouraging pharmacies from offering these new products to their customers. Pharmacists will require additional education through future digital health certification programs in order to counsel patients and caregivers about OTC DTx and PDTs being offered in their pharmacies [90,223,224]. The International Pharmaceutical Federation reported that many pharmacy and pharmaceutical education programs lack digital health education [130], while the American Association of Colleges of Pharmacy recognizes needs to integrate digital health education into the PharmD curriculum [225]. Currently, pharmacists can utilize educational resources provided by the Digital Therapeutics Alliance, the Digital Medicine Society, and the Digital.Health platform, as well as receive certifications from several digital health online courses. 

For store-within-a-store pharmacy operations, it would be difficult to eliminate SSB, alcohol, and tobacco sales, since such retail spaces sell food, beverages, and prescription drugs under one roof. In such circumstances, shelves containing SSBs, alcohol, and tobacco can also display information about health risks associated with these products [226,227,228,229]. California has a policy requiring point-of-sale warnings that exposure to alcohol increases the risk of cancer, while Ireland is the first country that requires informing consumers about “the direct link between alcohol and fatal cancers” using health warning labels [230,231]. Discontinuing sales of health-harming products is also associated with challenges like financial interests (commercial determinants of health and lobbying) that may interfere with a timely response to the growing evidence of the negative impact of SSBs on individual and public health [232,233,234,235]. 

Navigating the major transformation of pharmacy practice was delineated in the report on “Catalyzing Action for Pharmacist-Provided Food Is Medicine Care” published by the Milken Institute [186]. It is reasonable to extend such transformation strategy towards “Pharmacist-Provided Digital Health Care”, resulting in more integrative pharmacy care that can improve the mortality and morbidity rates in the US and worldwide. The foremost changes require aligning interests of all stakeholders, including pharmacy leadership, healthcare systems, advocacy groups, trade associations, the digital health industry, healthcare providers, public health professionals, investors, caregivers, and patients. 

## 8. Conclusions

There is a need to address a costly paradox and discrepancy between the highest healthcare spending per capita and the highest rates of avoidable mortality in the US compared to other high-income countries. Drug discovery, development of DTx, and other medical innovations are insufficient to combat chronic diseases, unless there are concurrent efforts to reduce their preventable and self-inflicted causes. To this end, our perspective article describes a rationale for redesigning retail pharmacy spaces to improve US healthcare outcomes by trading sales of health-harming products with health-promoting digital technologies. 

“As healthcare systems work under increasingly dynamic and resource-constrained conditions, evidence-based strategies are essential to ensure that research investments maximize healthcare value and improve public health” [236]. A growing body of evidence challenges pharmacies to reach a decision point of either continuing or discontinuing sales of SSBs, due to the increased risks for premature death, cancer, CVDs, diabetes, and other chronic diseases. Like for tobacco and alcohol products, there is no rationale for SSBs to be offered and promoted through pricing in pharmacies. At the same time, a growing body of evidence on clinically meaningful benefits of digital health technologies offers pharmacies opportunities to expand their revenue sources and profit margins by offering digital therapeutics (OTC DTx and PDT), mobile apps, and wearables supporting disease self-management and healthy lifestyles. Market competition, self-regulation, and growing public awareness about the pros and cons of digital health tech versus SSBs will determine how quickly retail pharmacies will evolve, adopt, and promote digital transformation towards precision healthcare [45,237,238,239,240]. 

## Figures and Tables

**Table 2:** An evidence-based rationale for eliminating sales and marketing of sugar-sweetened beverages in healthcare spaces, such as retail pharmacies. Examples of studies that show effects of SSBs on mortality and morbidity rates.

Studies	Main Findings
Malik et al. [156]	A positive association between consumption of SSBs and breast cancer mortality. Women who consume more than two servings of SSBs/day are at higher risk of death
Mullee et al. [157]	Higher mortality was found among participants who consumed more than two servings of SSBs/day. A positive association between consumption of SSBs and death from circulatory diseases
Alcaraz et al. [158]	More than 184,000 deaths per year worldwide are attributed to SSB consumption
Yuan et al. [159]	Higher SSB consumption positively correlated to proximal colon cancer mortality. SSB consumption enhances colorectal tumorigenesis
Liu et al. [173] Chen et al. [174]	Higher SSB consumption associated with increased risk of developing Alzheimer’s disease
McCullough et al. [160]	SSB consumption was positively associated with the risk of colorectal cancer
Cordova et al. [175]	Ultraprocessed food consumption is associated with a higher incidence of multimorbidity. Type 2 diabetes and cardiovascular diseases are more prevalent among consumers of ultraprocessed foods
Malik et al. [165]	Consumption of SSBs promotes obesity in children and adults
Wang et al. [168], Li et al. [176]	Higher consumption of SSBs is associated with higher risks for type 2 diabetes
Jiantao et al. [177]	Consumption of SSBs is associated with a higher risk of developing prediabetes and insulin resistance
Wang et al. [170], Danqing et al. [169]	Dose-dependent consumption of SSBs increases risks for depression

## Data Availability

No new data were created or analyzed in this study. Data sharing is not applicable to this article.

## Supplementary Materials

The following supporting information can be downloaded at https://www.mdpi.com/article/10.3390/pharmacy12040107/s1: Figure S1: An example of alcohol aisles in a national retail pharmacy in the US; Figure S2: The number of sugar-sweetened beverages sold through 75 pharmacies belonging to a national pharmacy chain. The plot was generated based on data published in [154]; Table S1: CHRs from five different review articles used to create a graph shown in Figure 4.
