# Supplementary material for: Redesigning Pharmacy to Improve Public Health Outcomes: Expanding Retail Spaces for Digital Therapeutics to Replace Consumer Products That Increase Mortality and Morbidity Risks
Source: Pharmacy (Basel). 2024 Jul 12;12(4):107. doi: 10.3390/pharmacy12040107 (PMC11270305; doi:10.3390/pharmacy12040107)

pharmacy

MDPI

Supplementary Materials

Redesigning Pharmacy to Improve Public Health Outcomes: Expanding Retail Spaces for Digital Therapeutics to Replace Consumer Products That Increase Mortality and Morbidity Risks

Grzegorz Bulaj [1,*], Melissa Coleman [2], Blake Johansen [2], Sarah Kraft [3], Wayne Lam [2], Katie Phillips [2] and Aarushi Rohaj [1,4]

[1] Department of Medicinal Chemistry, College of Pharmacy, University of Utah, Salt Lake City, UT 84112, USA
[2] College of Pharmacy, University of Utah, Salt Lake City, UT 84112, USA
[3] Independent Researcher, Salt Lake City, UT 84112, USA
[4] The Spencer Fox Eccles School of Medicine, University of Utah, Salt Lake City, UT 84112, USA
* Correspondence: bulaj@pharm.utah.edu

Table S1. CHRs from five different articles show above 1 means that drinking more than 2 SSBs daily will more likely contribute to the hazard state when compared to the control of drinking less than 1 SSB per month.

Articles	Studies	Sample Size	Hazard States	Observation/ Control	95% CI	Cox's Ratio
Malik et al., Circulation, 2019	NHS	80,647	death	M2D/1DM	1.52,1.75	1.63
	HPFS	37,716	death	M2D/1DM	1.15,1.44	1.29
	NHS	80,647	breast cancer	M2D/1DM	1.00,1.80	1.34
Mullee et al., JAMA Internal Medicine, 2019	EPIC	451,743	death	M2D/1DM	1.11,1.22	1.17
	EPIC	451,743	circulatory dis- eases	M2D/1DM	1.30,1.78	1.52
	EPIC	451,743	digestive diseases	M2D/1DM	1.24,2.05	1.59
Yuan et al., Am J Clin Nutr, 2022	*NHS, HPFS	121,111	proximal colon cancer incident	M2D/0D	1.01,1.88	1.38
	*NHS, HPFS	121,111	proximal colon cancer death	M2D/0D	1.28,3.43	2.09
McCullough et al, Cancer Epi- demiol Biomarkers Prev, 2022	ACS	1,184,284	colorectal cancer	M2D/0D	1.02,117	1.09
	ACS	1,184,284	kidney cancer	M2D/0D	1.03,1.34	1.17
	ACS	1,184,284	Non-Hodgkin Lymphoma	M2D/0D	1.11,1.41	1.25
Cordova et at, Lancet Reg Health, 2023		451,743	Multi- morbidity	MT/No UPF	1.05,1.12	1.09

FOOTNOTES:.

Nurses' Health Study (NHS) 1980-2014, women.

Health Professional's Follow-up (HPFS)1986-2014, men.

European Prospective into Cancer & Nutrition (EPIC) 1992-2000, men & women.

*NHS (1984-2014) and HPFS (1986-2014), men & women.

American Cancer Society (ACS) 1982, men & women.

more than 2 drinks per day (M2D) 1 drink per month (1DM).

0 drinks per month (OD).

more than 413g for men & 326g for women per day (MT) Ultra-Processed Food (UPF)

Figure S1. An example of alcohol aisles in a national retail pharmacy in the US.

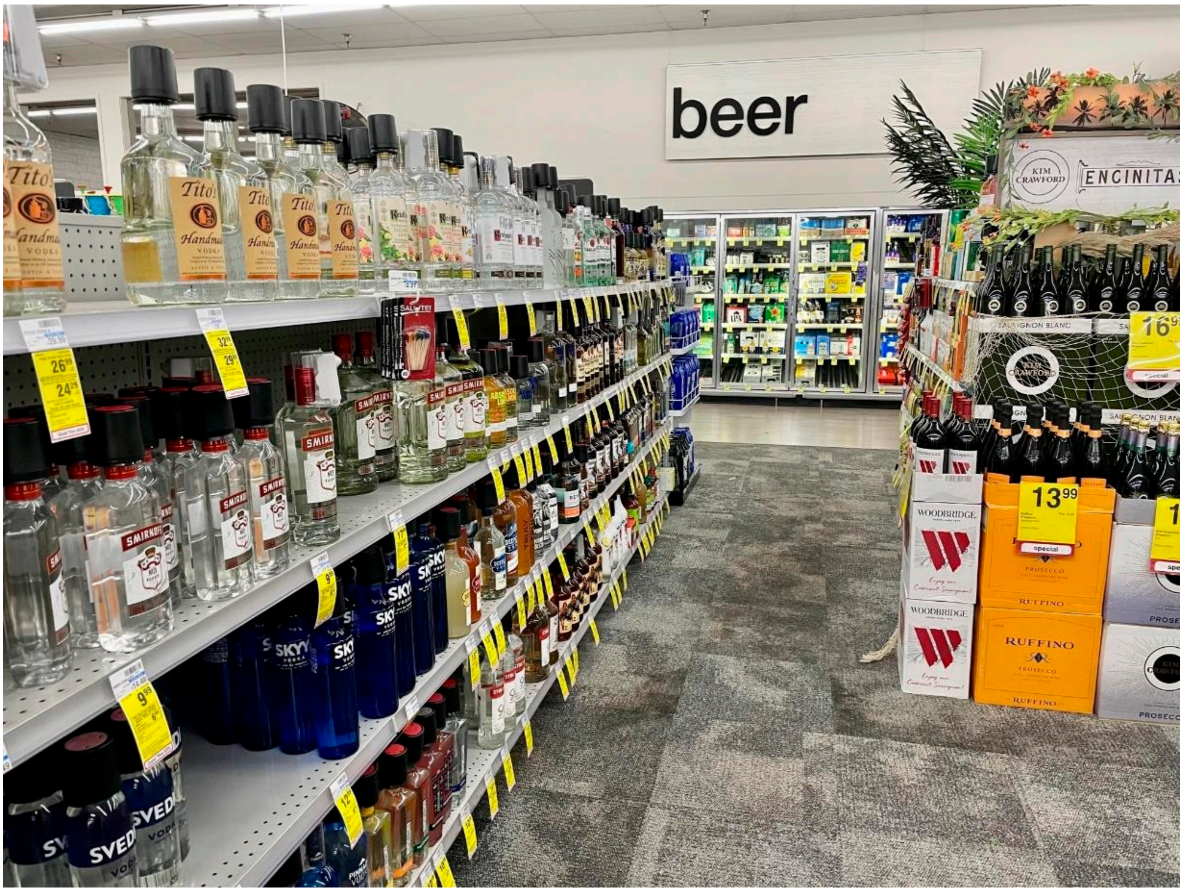

Figure S2. A number of sugar-sweetened beverages sold through 75 pharmacies belonging to a national pharmacy chain. The plot was generated based data published in Hua et al, (2023) *Philadelphia Beverage Tax and Association With Prices, Purchasing, and Individual-Level Substitution in a National Pharmacy Chain,* **JAMA Network Open**. Vol. 6 Issue 7 Pages e2323200-e2323200. DOI: 10.1001/jamanetworkopen.2023.23200.

The published data from Hua et al suggest that 75 pharmacy stores sold approximately 447,000 soda drinks per year (2017 data), yielding 5,590 SSB transactions per store per year. To estimate SSB transactions throughout two major pharmacy chains in the US, the number of chain pharmacy stores (CVS Pharmacy 9,169; Walgreens 8,250; combined 17,410 stores; 2024 data) was multiplied by 5,590. 17,410 pharmacy stores (x 5,590 SSB per store per year) can lead to sales reaching approx. 100 million soda beverages per year in the US. (as a reference in 2022, the US population was 333 million).

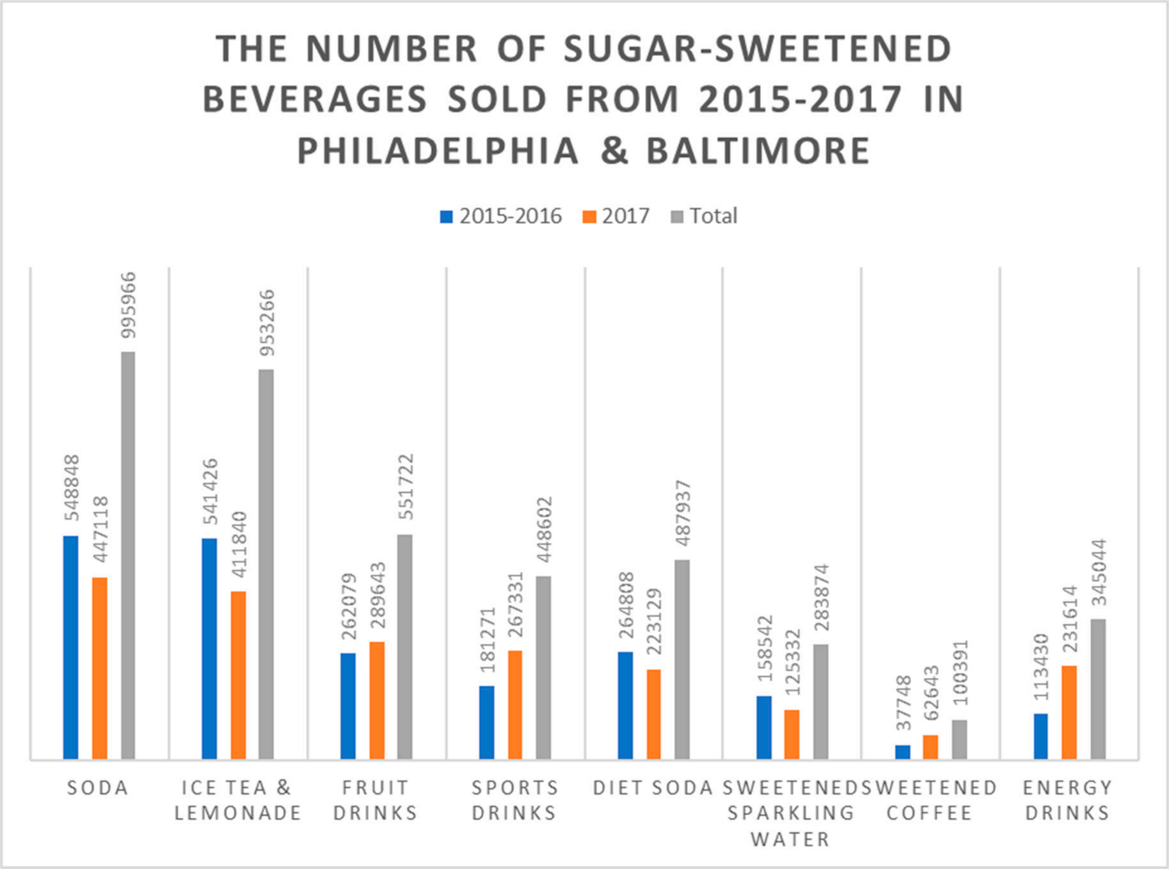

Supplement: Supplementary file 1 [file pharmacy-12-00107-s001.zip › pharmacy-3058409-supplementary.pdf]
